# The factors associated with the modified Fisher grade in patients with aneurysmal subarachnoid hemorrhage

**DOI:** 10.3389/fphys.2024.1373925

**Published:** 2024-07-17

**Authors:** Di Zhao, Yating Li, Jianzhong Cui

**Affiliations:** ^1^ Department of Neurosurgery, The Fourth Hospital of Hebei Medical University, Shijiazhuang, China; ^2^ School of Nursing, Hebei Medical University, Shijiazhuang, China; ^3^ Department of Surgery, Hebei Medical University, Shijiazhuang, China; ^4^ Department of Neurosurgery, Tangshan Gongren Hospital, Tangshan, China

**Keywords:** aneurysmal subarachnoid hemorrhage, intracranial hemorrhage, Fisher grade, risk factor, machine learning approach

## Abstract

**Background:**

Aneurysmal subarachnoid hemorrhage (aSAH) is a life-threatening medical condition with a high fatality and morbidity rate. There was a substantial link between the modified Fisher grade of aSAH and the neurological function deficit. This study aimed to analyze the factors associated with the modified Fisher grade of aSAH using a machine learning approach.

**Methods:**

A multi-center observational study was conducted. The patients with aSAH were recruited from five tertiary hospitals in China. The volume of hemorrhage in aSAH was measured using the modified Fisher grade scale. The risk factors responsible for the modified Fisher grade of aSAH were analyzed, which include sociodemographic factors, clinical factors, blood index, and ruptured aneurysm characteristics. We built several tree-based machine learning models (XGBoost, CatBoost, LightGBM) for prediction and used grid search to optimize model parameters. To comprehensively evaluate the model, we used Accuracy, Precision, Area Under the Receiver Operating Characteristic Curve (AUROC), Area Under the Precision-Recall Curve (AUPRC), and Brier as evaluation indicators to assess the model performance and select the best model.

**Results:**

A total of 888 patients with aSAH were recruited, of whom 305 with modified Fisher grade of 3 and 4. The results show that the XGBoost model has the highest AUROC of 0.772, and the indicators are better than CatBoost and LightGBM. The feature importance graph shows that the top feature variables include platelet, thrombin time, fibrinogen, preadmission systolic blood pressure, activated partial thromboplastin time, and the time interval between the onset of aSAH and the first-time CT examination.

**Conclusion:**

The factors responsible for the modified Fisher grade of aSAH were identified, which offered valuable insights for future research and clinical intervention. These risk factors should be controlled in the treatment of unruptured aneurysms, and appropriate treatment can be given if necessary to reduce the risk of severe hemorrhage after aneurysm rupture.

## 1 Background

Aneurysmal subarachnoid hemorrhage (aSAH) is a serious neurological emergency caused by the accumulation of blood in the subarachnoid space as a result of a ruptured aneurysm (Ran et al., 2023). According to statistics, the incidence of aSAH reached 9–11 per 100,000 people/year worldwide (Shen et al., 2021). Improvements in the clinical management of aSAH have resulted in better patient outcomes during the last several decades. aSAH, on the other hand, is still associated with significant morbidity and mortality (Daou et al., 2019; Neifert et al., 2021). According to recent studies, aSAH has a 30-day case mortality of about 20% and a functional dependency rate of up to 50% among survivors(Ran et al., 2023). The long-term sequelae of aSAH have a significant impact on public health, leading to reduced quality of life for patients (Claassen and Park, 2022). The modified Fisher grade of aSAH is closely related to the development of functional deficits as well as mortality. It is also considered an important clinical and research tool for predicting neurological complications of aSAH (Páez-Granda et al., 2021). Therefore, it is important to determine the risk factors associated with the modified Fisher grade of aSAH (Eagles et al., 2018).

The previous single-center study conducted in 2016 by our research team has provided preliminary results about the sociodemographic factors, clinical factors, and ruptured aneurysm characteristics associated with the modified Fisher grade of aSAH and the results showed that preadmission diastolic blood pressure, multiple aneurysms, and aneurysms of the anterior communicating artery are associated with markedly increased modified Fisher grades (Liu et al., 2016). In this current study, more associated risk factors, especially the blood coagulation index was added to the analyzed factors. Additionally, the current study is a multi-center study that involves five centers, all of which are tertiary hospitals.

The machine learning approach is a promising approach to analyze factors associated with the modified Fisher grade of aSAH because it can offer quick, immediate, and accurate identification of risk factors (Deo, 2015). A tree-based integrated model was used to predict the model, and the optimal model was used to analyze the risk factors, thereby assisting clinical decision-making (Kim et al., 2021).

## 2 Materials and methods

### 2.1 Participants

A multi-center retrospective observational study was conducted. The participants were included from five hospitals in China from January 2020 to January 2023. All hospitals were tertiary hospitals with more than 2,000 beds. The inclusion criteria include 1) patients with spontaneous subarachnoid hemorrhage; 2) diagnosis of intracranial aneurysm; 3) modified Fisher grade scale ≥1; 4) received DSA or CTA examination to obtain aneurysmal-related data. The exclusion criteria include 1) Non-aneurysmal subarachnoid hemorrhage; 2) The majority of study variables that could not be obtained.

### 2.2 Variables

The study explored factors associated with the modified Fisher grade of aSAH, including sociodemographic factors (i.e., gender, age, smoking, drinking), clinical factors (i.e., hypertension, diabetes mellitus, hyperlipidemia, family history of cerebrovascular disease, history of drug or food allergies, whether or not use drug treatment on blood pressure, preadmission systolic blood, preadmission diastolic blood pressure, pulse pressure, the time interval between the onset of aSAH and the first-time CT examination), blood index (elevated blood lipids, elevated uric acid, hemoglobin [Hb], Red blood cell specific volume [Hct], blood platelet [Plt], Prothrombin time [PT], International Normalized Ratio [INR], Activated Partial Thromboplastin Time [APTT], thrombin time [TT], fibrinogen [Fib]), ruptured aneurysm characteristics (i.e., architecture, size, site, number of aneurysms, maximum lumen size of the aneurysms, aneurysmal neck length, and dome/neck ratio, history of ruptured aneurysm bleeding, whether or not recurrent bleeding before surgery). These variables were selected based on the recommendations from previous studies and clinical investigations.

The modified Fisher grade is a five-rank ordinal radiographic, CT-based measure that is calculated as follows: 0, no visualized SAH or intraventricular hemorrhage (IVH); 1, thin (<1 mm) SAH with no IVH; 2, thin SAH with IVH; 3, thick (≥1 mm) SAH with no IVH; and 4, thick SAH with IVH (Oliveira et al., 2011). The modified Fisher grade was assessed at the time of hospital admission. In this study, it is defined that a modified Fisher grade equal to or less than 2 as low grade of hemorrhage; whereas a modified Fisher grade of 3 and 4 as high grade of hemorrhage.

### 2.3 Statistical analysis

**FIGURE 1:**
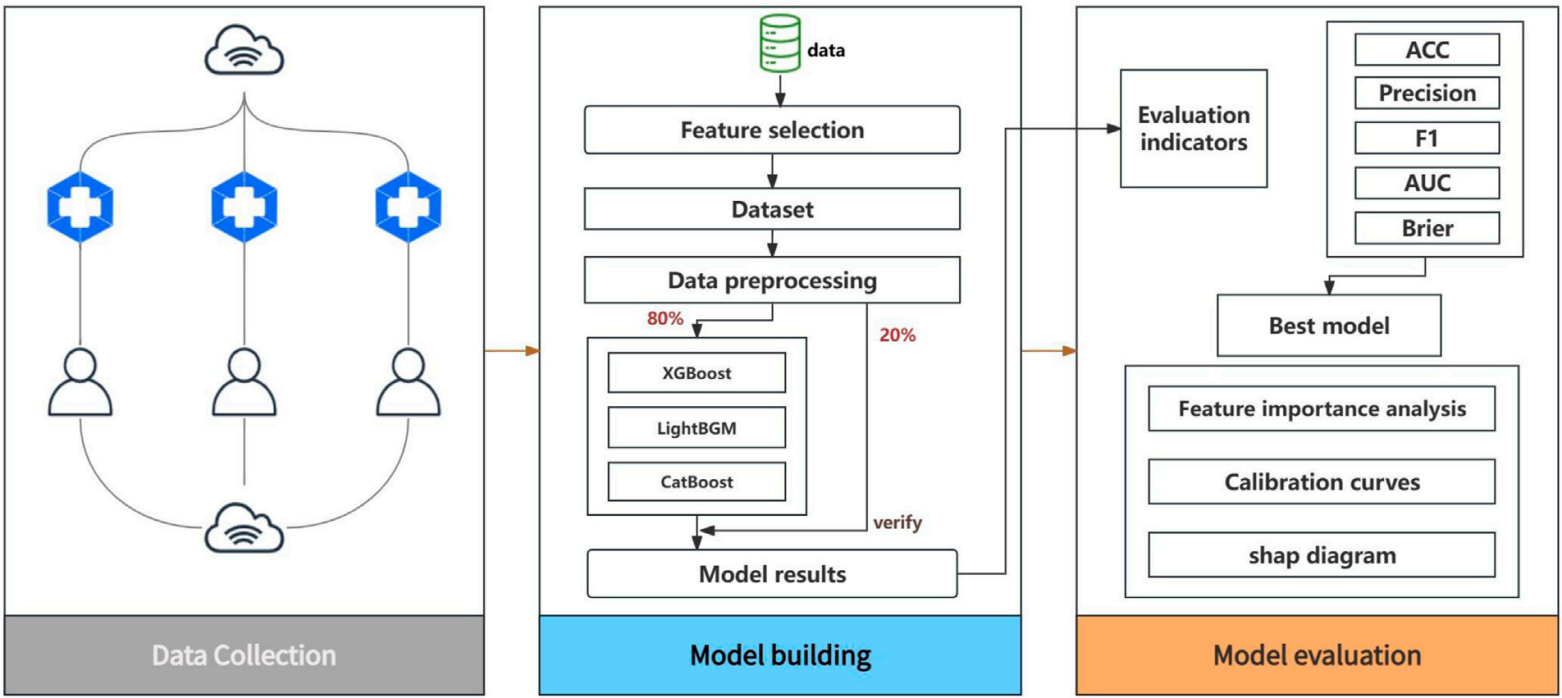
Model building and evaluation.

In this study, we used SPSS 26 and Python 3.9 as the main statistical analysis tools to ensure the accuracy and efficiency of the study. As shown in Figure 1, the process covers pre-processing, model development, model evaluation, and results analysis. The data is divided into an 80% training set and a 20% test set into the model.

In the pre-processing stage, in order to improve the computational efficiency of the model, feature screening is adopted to ensure that the model ultimately relies on the most predictive features and avoid the risk of over-fitting. Feature elimination based on random forest is adopted, and the feature space is effectively reduced by constructing a random forest model and gradually removing the features that contribute the least to the prediction. This method relies on the ability of random forests to evaluate the importance of each feature and selects the subset of features that are most closely associated with the target variable through a repeated iterative process.

For model development, gradient boosting algorithms based on tree models were used: XGBoost, CatBoost, and LightGBM (Chen et al., 2023; Fujihara et al., 2023). These models were chosen in part because they have unique and effective mechanisms for handling missing values, which can optimize the learning process and prediction ability of the model. When conducting data collection and data analysis, it is often encountered that there are unknown option on some items, which is usually regarded as a missing value in the data preprocessing. A common method for handling categorical variable missing values is to fill them in with modal values, but this method has its drawbacks, such as potentially leading to data bias, especially when the modal value is not very high. In contrast, using algorithms to handle missing values can be more flexible and precise. CatBoost assigns a specific path to missing values during model training, rather than simply ignoring them or filling them in with statistical methods (such as mean filling), which can more accurately reflect the actual distribution of the data and also extract valuable information from the missing values themselves. XGBoost adopts a default direction strategy, that is, when the decision tree is split, if the data is missing on a certain feature, the algorithm will automatically learn and choose an optimal direction to deal with these missing values, thus ensuring the maximum utilization of information. The processing method of LightGBM is to directly ignore the missing value when constructing the decision tree, wait until an optimal split point is found, and then classify the missing value to the most favorable branch according to the information gain. This method not only saves the cost of pre-processing the missing value, but also avoids the potential impact on the model prediction performance.

To comprehensively assess and compare the comprehensive performance of the different models, we used a series of key assessment indicators. These include accuracy (ACC) to measure the overall prediction correctness of the model, precision to evaluate the accuracy of the model in identifying positive cases, Area Under the Receiver Operating Characteristic Curve (AUROC) to assess the classification efficiency of the model at different thresholds, Area Under the Precision-Recall Curve (AUPRC) to evaluate model performance with fewer positive samples, Brier Score to quantify the accuracy of prediction probability, and the calibration curves to assess the reliability of the model predictions and the calibration level of the predicted probabilities.

### 2.4 Ethical consideration

The study received ethical approval from the ethics committee of the Fourth Hospital of Hebei Medical University.

## 3 Results

### 3.1 Participant characteristics

A total of 888 participants were included in this study. Table 1 provides the characteristics of the study participants. The mean age of the patients was 57.23 ± 12.16 years. 4.95% of the patients had recurrent bleeding before surgery, half of the patients had hypertension, 22.86% had a history of smoking, 16.67% had a history of drinking.

**TABLE 1:** Characteristics of participants.

Variables		Frequency (n = 888)	Percentage(%)
Gender			
	woman	557	62.72
	man	331	37.28
Hypertension			
	yes	442	49.77
	no	443	49.88
	unknown	3	0.35
Diabetes			
	no	811	91.33
	yes	73	8.22
	unknown	4	0.45
Hyperlipidemia			
	no	738	83.10
	unknown	127	14.30
	yes	23	2.60
Smoking			
	no	678	76.32
	yes	203	22.86
	unknown	7	0.82
Drinking			
	no	737	83.00
	yes	148	16.67
	unknown	3	0.33
History of drug or food allergies			
	no	876	98.65
	yes	10	1.11
	unknown	2	0.24
History of ruptured aneurysm bleeding			
	no	854	96.17
	yes	34	3.83
Family history of cerebrovascular disease			
	no	857	96.50
	yes	16	1.80
	unknown	15	1.70
Drug treatment on blood pressure			
	no	453	51.01
	yes	344	38.74
	unknown	91	10.25
Recurrent bleeding before surgery			
	no	844	95.05
	yes	44	4.95
Elevated uric acid			
	no	624	70.27
	unknown	213	23.99
	yes	51	5.74
Aneurysm architecture			
	Saccular aneurysm	690	77.70
	Lobulated aneurysm	76	8.56
	Fusiform aneurysm	52	5.86
	Dissecting aneurysm	35	3.94
	Pseudoaneurysm	19	2.14
	Blister aneurysm	16	1.80
Aneurysm number			
	one	700	78.83
	two	150	16.89
	three	24	2.70
	≥four	14	1.58
Aneurysm site			
	Anterior circulation aneurysm	792	89.19
	Posterior circulation aneurysm	96	10.81
modified Fisher grade			
	≤2	583	65.65
	≥3	305	34.35

Variables	Mean ± SD/Median (IQR)
Age	57.23 ± 12.16
Preadmission systolic blood pressure (mmHg)	141.41 (26.25)
Preadmission diastolic blood pressure (mmHg)	82 (16.25)
Time interval between the onset of aSAH and the first-time CT examination (minutes)	1436.32 ± 3199.92
Hb (g/L)	131.10 ± 20.27
Hct	38.82 ± 5.28
Plt (10^9^/L)	227.87 ± 72.27
PT (seconds)	11.97 ± 1.25
INR	1.03 ± 0.13
APTT (seconds)	28.46 ± 4.48
TT (seconds)	15.83 ± 10.22
Fib (g/L)	3.24 ± 1.54
Maximum lumen size (mm)	5.16 ± 3.65
Neck length (mm)	3.49 ± 2.23

Note: SD, standard deviation; IQR, interquartile range; Hb, hemoglobin; Hct, Red blood cell specific volume; Plt, blood platelet; PT, prothrombin time; INR, international normalized ratio; APTT, activated partial thromboplastin time; TT, thrombin time; Fib, Fibrinogen.

Most of the patients had saccular aneurysm, accounting for 77.70%, while the proportion of blister aneurysm was the smallest, only 1.80%. 78.83% of patients suffered a single aneurysm. The aneurysm location was mainly anterior circulation aneurysm (89.19%).

### 3.2 Predicting results

**FIGURE 2:**
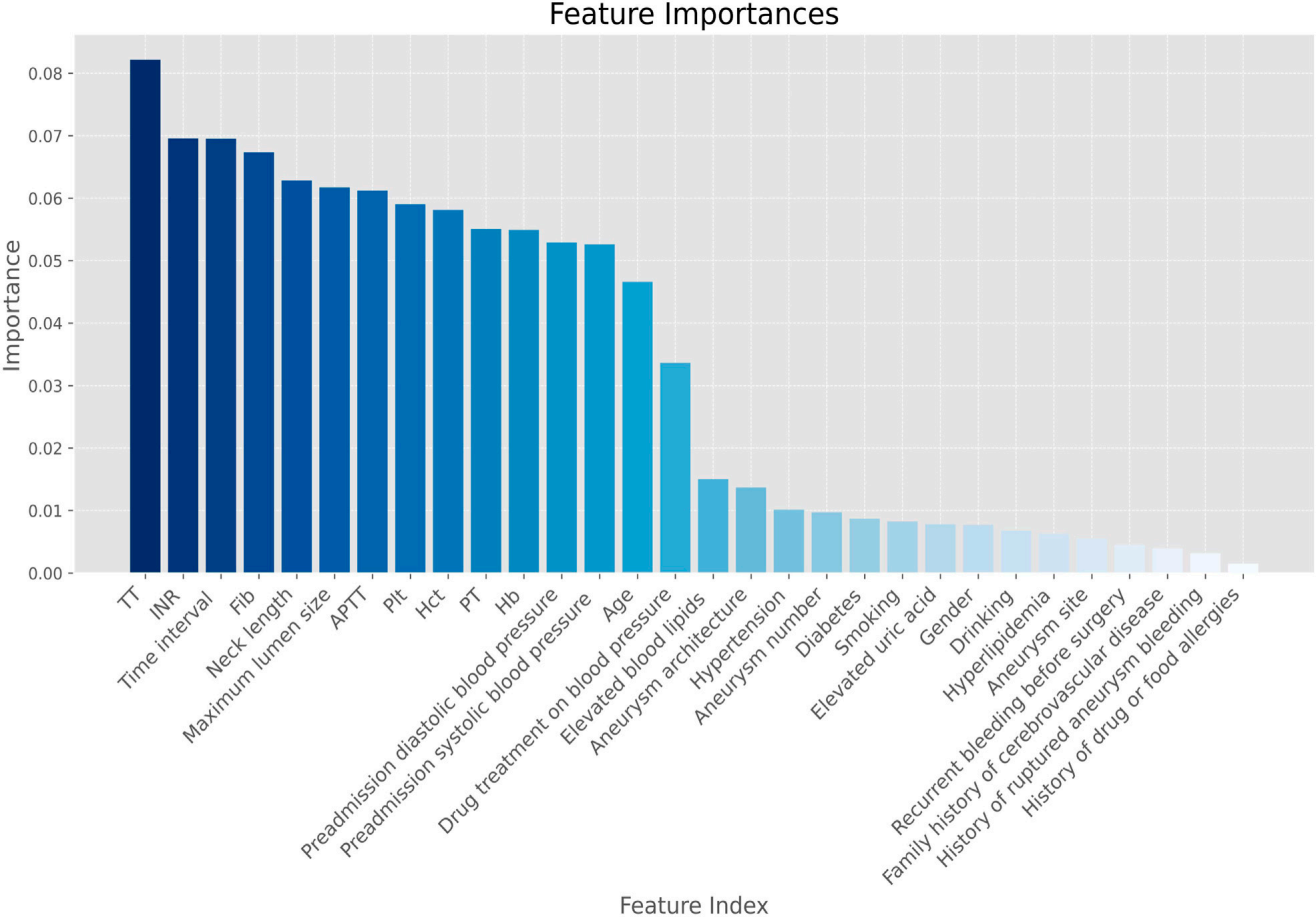
Feature screening importance plot. Note. Hb, hemoglobin; Hct, red blood cell specific volume; Plt, blood platelet; PT, prothrombin time; INR, international normalized ratio; APTT, activated partial thromboplastin time; Time interval, time interval between the onset of aSAH and the first-time CT examination; TT, thrombin time; Fib, Fibrinogen.

By random forest we successfully screened the 14 most influential features from a dataset containing 30 features by using a feature selection method, with 0.2 as a threshold (Figure 2).

**FIGURE 3:**
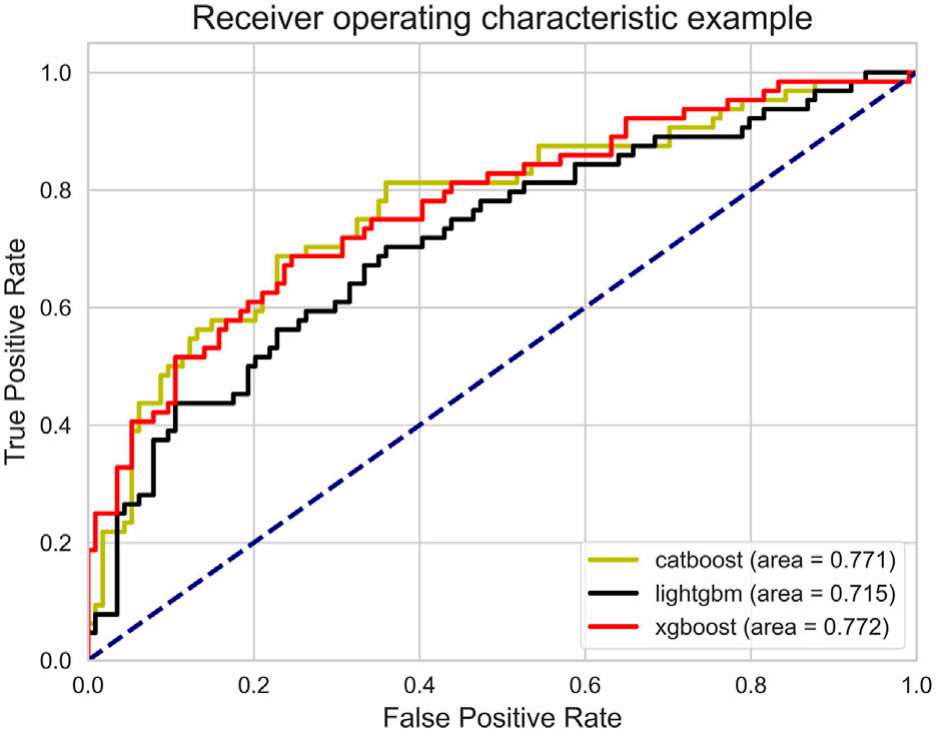
Receiver operating characteristic.

The AUROC of the three models is shown in Figure 3. Ideally, the ROC curve of the model is close to the upper left corner, indicating the high true case rate and the low false positive case rate. In this figure, XGBoost (AUROC = 0.772) has a slightly higher AUROC value, followed by CatBoost (AUROC = 0.771) and LightGBM (AUROC = 0.715). This means that XGBoost performs best in balancing true and false positive cases.

After thoroughly analyzing the comprehensive performance metrics in Table 2, we observed that the CatBoost model slightly outperformed the other models on ACC and Precision, suggesting that CatBoost is more accurate in positive class prediction. However, it is also seen from the table that XGBoost outperformed the other models in AUROC, AUPRC and Brier. The high value of AUROC indicates the overall advantage of XGBoost in distinguishing positive and negative categories, while its performance on AUPRC illustrates the accuracy of model positive class prediction in unbalanced datasets. On the other hand, the lower Brier scores highlight the accuracy and reliability of the XGBoost prediction probability, meaning the possibility that its prediction results are closer to the real situation. These results collectively show that XGBoost provides a more balanced and robust performance in overall prediction performance. Therefore, the XGBoost model was secelted as the optimal model.

**TABLE 2:** Comparison of predicted performance.

	ACC	Precision	AUROC	AUPRC	Brier
XGBoost	0.736	0.743	**0.772**	**0.710**	**0.180**
CatBoost	**0.747**	**0.757**	0.771	0.682	0.184
LightGBM	0.702	0.622	0.715	0.606	0.199

Note: ACC, accuracy; AUROC, area under the receiver operating characteristic curve; AUPRC, area under the precision-recall curve.

**FIGURE 4:**
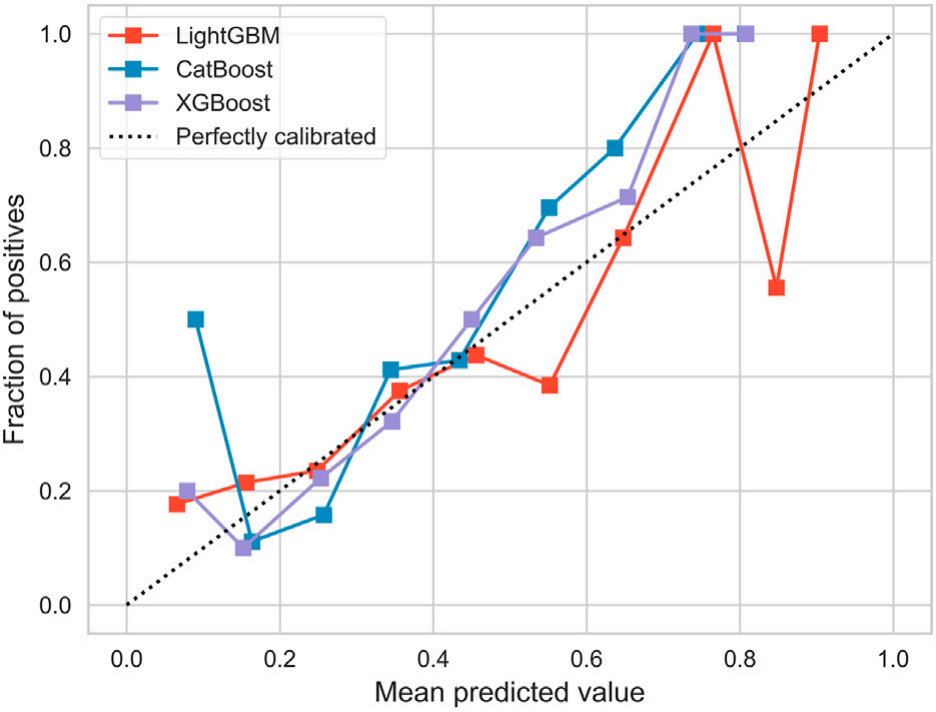
Calibration curve.

Figure 4 illustrates the overall calibration curves of the three models. The calibration curve shows the change in performance for each model, compared to the perfect agreement between the predicted and actual probabilities of the model. The ideal calibration curve (expressed as the dotted black dot), along the diagonal, means that the predicted probability perfectly corresponds to the probability of actual occurrence. In this figure, the calibration curves of all models deviate from the ideal line, and LightGBM deviates the most significantly in the region with higher prediction probability, while the curves of CatBoost and XGBoost are close to the ideal line, especially in the region of moderate probability values. However, in the high-probability regions, the calibration curves of CatBoost and XGBoost fluctuate greatly, indicating an unstable prediction accuracy in these regions. Overall, the calibration curve of XGBoost performed best.

### 3.3 Feature importance map

XGBoost’s feature importance graph is based on a tree model and measures the importance of features in the model. The contribution of each feature to the model prediction is visualized by calculating the weight of the number of times the feature is used for splitting, the average gain resulting from the feature splitting, and the sample coverage during the feature splitting process.

**FIGURE 5:**
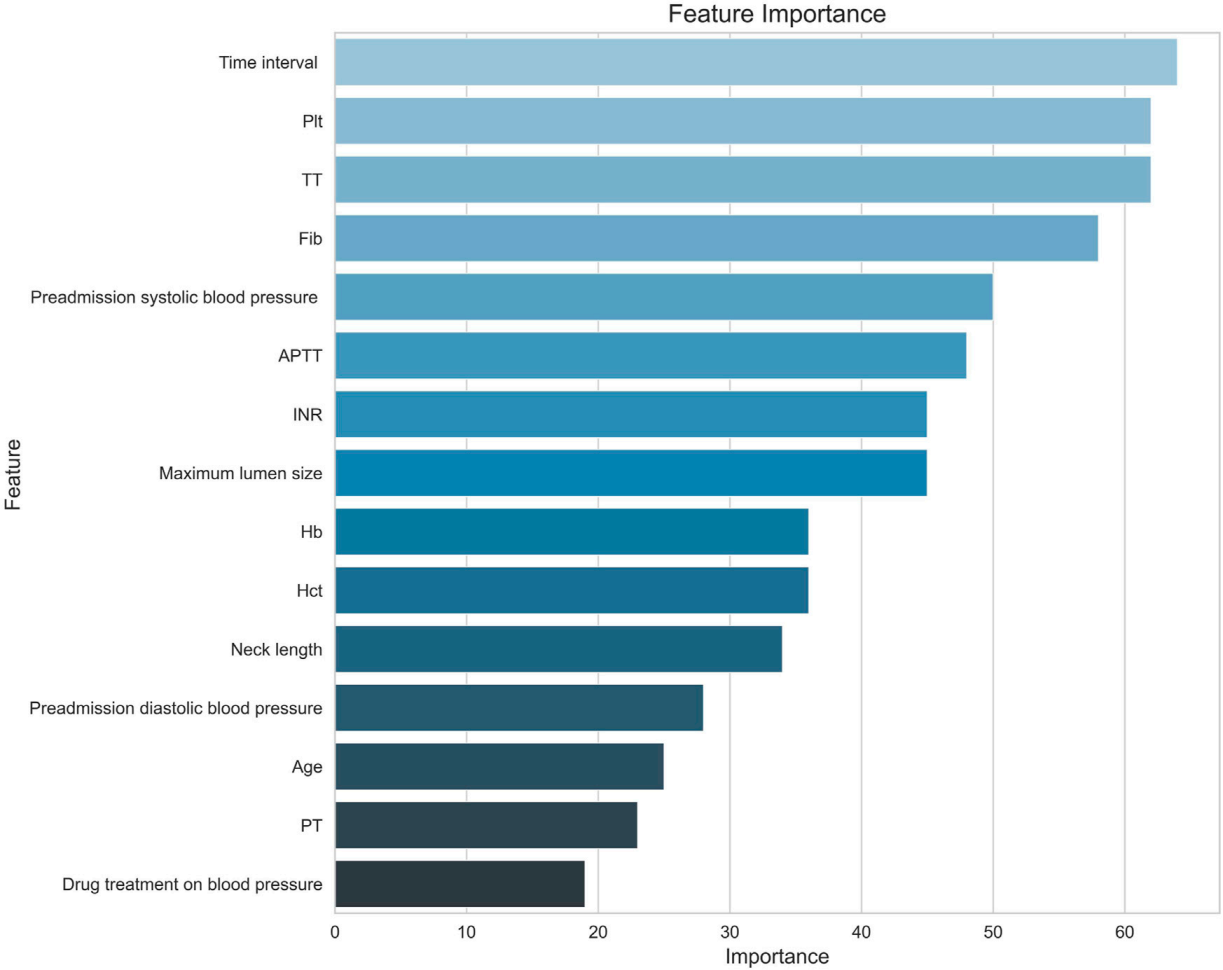
Feature importance diagram. Note. Hb, hemoglobin; Hct, red blood cell specific volume; Plt, blood platelet; PT, prothrombin time; INR, international normalized ratio; APTT, activated partial thromboplastin Time; TT, thrombin time; Fib, fibrinogen; Time interval, the time interval between the onset of aSAH and the first-time CT examination.

As can be seen from the feature importance graph (Figure 5), the time interval between the onset of aSAH and the first-time CT examination, platelet, thrombin time, fibrinogen, preadmission systolic blood pressure, and activated partial thromboplastin time are the top risk factors that associated with the modified Fisher grade of aSAH.

**FIGURE 6:**
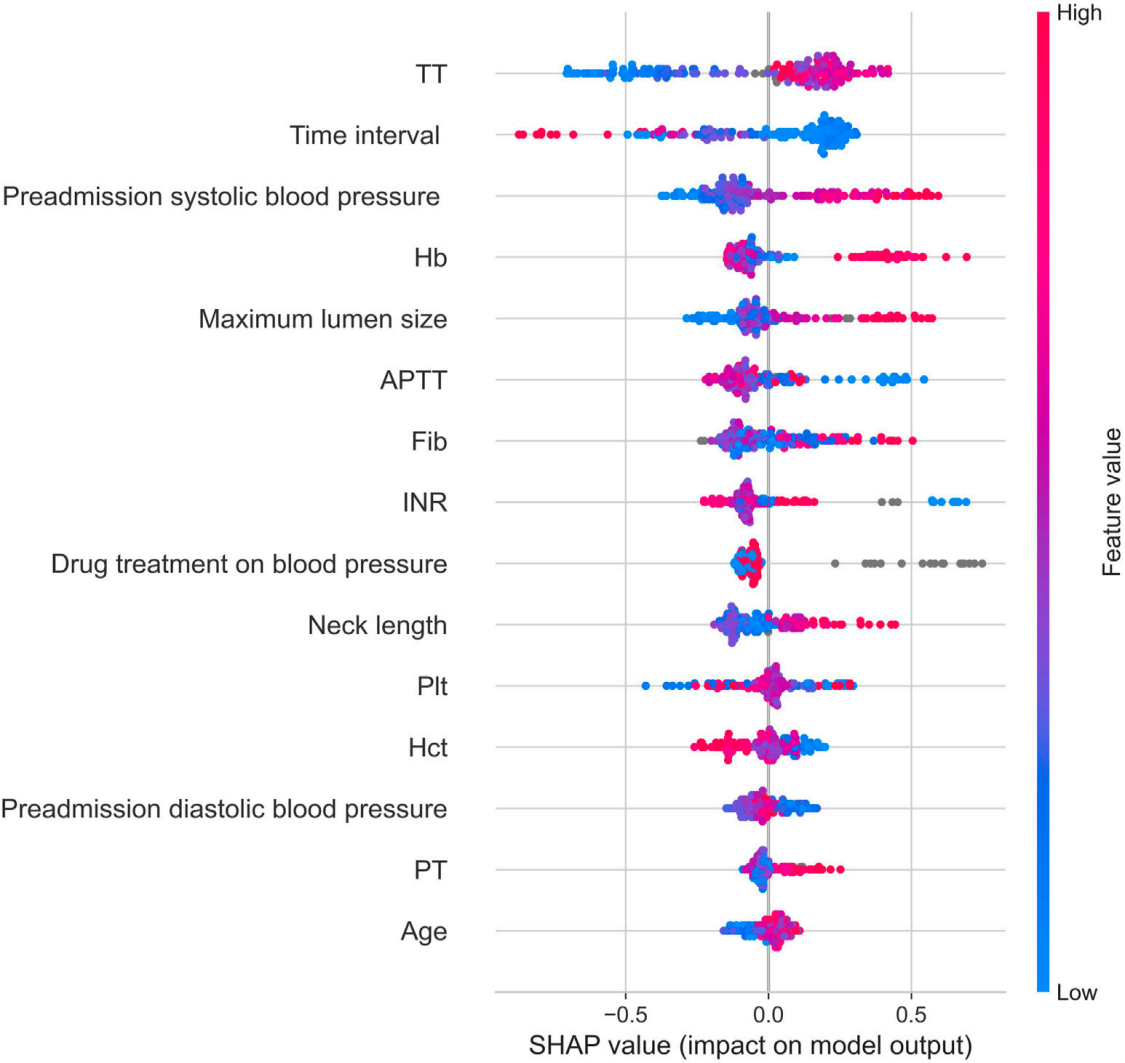
Shap graph. Note: Hb, hemoglobin; Hct, red blood cell specific volume; Plt, blood platelet; PT, prothrombin time; INR, international normalized ratio; APTT, activated partial thromboplastin Time; TT, thrombin time; Fib, fibrinogen; Time interval, the time interval between the onset of aSAH and the first-time CT examination.

Shapley Additive Explanations Plots (SHAP plots) provide interpretability of the importance of features in the XGBoost model. It is based on the Shapley value of game theory, quantifying the average contribution of each feature to the prediction. The SHAP plot shows the direction and magnitude of each feature’s influence, helping to understand the model’s decision-making process. As shown in Figure 6, thrombin time and neck length are positively correlated with the modified Fisher grade of aSAH, while the time interval between the onset of aSAH and the first-time CT examination and activated partial thromboplastin time are negatively correlated with the modified Fisher grade of aSAH.

## 4 Discussion

The aSAH is a common hemorrhagic cerebrovascular disease, causing devastating damage not only to the central nervous system, but also to many other organs. aSAH is associated with significant morbidity and mortality despite advances in care and aneurysm treatment strategies (Virta et al., 2022; Ran et al., 2023). There is a close relationship between the modified Fisher grade of aSAH and the development of functional deficits as well as mortality in the patients. Identifying the risk factors associated with the modified Fisher grade of aSAH is important. This study combined multiple variables to build machine learning models, the patient’s sociodemographic factors, clinical factors, blood index, and ruptured aneurysm characteristics were incorporated into the model to analyze the factors associated with the modified Fisher grade of aSAH. XGBoost achieved the AUROC of 0.772, which is generally better than other machine learning models and shows good predictive performance.

The factors responsible for the modified Fisher grade of aSAH were identified. The feature importance curve shows that the top feature variables include the time interval between the onset of aSAH and the first-time CT examination, platelet, thrombin time, fibrinogen, preadmission systolic blood pressure, and activated partial thromboplastin time. This study offered valuable insights for clinical intervention and future research.

This study showed that the time interval between the onset of aSAH and the first-time CT examination was associated with the modified Fisher grade of aSAH. Patients with aSAH may exhibit clinical symptoms such as headache, nausea, vomiting, visual disturbances, and altered consciousness. Ignoring symptoms or delaying diagnosis can lead to serious consequences, including death and severe disability. Non-enhanced head CT remains the primary diagnostic tool for aSAH. Clinical staff should optimize workflow and perform CT examinations on symptomatic patients as early as possible for early diagnosis and treatment (Hoh et al., 2023).

Platelets are also associated with the modified Fisher grade of aSAH. Platelets are the blood cells responsible for blood coagulation and hemostasis. Typically, an increase in platelet values may increase the risk of blood clots forming. Low platelet values may lead to decreased hemostasis and increased risk of bleeding (He et al., 2019). Therefore, maintaining appropriate platelet values is essential to decrease modified Fisher grade and prevent the occurrence of complications in patients with aSAH.

The study indicated that fibrinogen, thrombin time, and activated partial thromboplastin time are related with the modified Fisher grade of aSAH. Fibrinogen is the main factor that determines plasma viscosity and plays an important role in regulating blood coagulation, fibrinolysis, inflammation and wound healing (May et al., 2021). The study by Xie (2020) also showed that the level of peripheral blood fibrinogen in patients with poor prognosis of aSAH was lower than that in patients with good prognosis, and the serum fibrinogen level could be used as a treatment target in patients with aSAH (Xie et al., 2020). Additionally, thrombin time measures the conversion of fibrinogen to fibrin, and the activated partial thromboplastin time is the most commonly used indictor to reflect whether the endogenous coagulation system is functioning normal or not (Santoro et al., 2023). These coagulation indicators are associated with the modified Fisher grade of aSAH, and should be paid attention to improve the prognosis of patients with aSAH (Li et al., 2023).

Furthermore, the findings showed that a high pre-admission systolic blood pressure contributed to modified Fisher grade of aSAH. Similarly, the previous study showed that high systolic blood pressure after aSAH has been related with an increased risk of rebleeding (Calviere et al., 2022). It is suggested that intensive systolic blood pressure lowering strategy be administrated for patients with aSAH to decrease the modified Fisher grade of aSAH (Etminan et al., 2019; Calviere et al., 2022; Ran et al., 2023).

There are some limitations in this study. The overall prediction accuracy of the model still has room for improvement. Future studies could consider using different model structures, or using other techniques to explore risk factors that influence the modified Fisher grade of aSAH. Additionally, future studies could consider introducing more potential variables, such as whether the subjects received anticoagulation intervention before surgery into the analyze (Claassen and Park, 2022). Nevertheless, this study identified the factors influencing the modified Fisher grade of aSAH, providing valuable insights into future research and clinical interventions. These risk factors should be paid attention to in the treatment of unruptured aneurysms, and appropriate intervention can be given if necessary to reduce the risk of severe bleeding after aneurysm rupture, so as to improve patient outcomes.

## Data Availability

The raw data supporting the conclusions of this article will be made available by the authors, without undue reservation.
